# Radiation Induces Acute Alterations in Neuronal Function

**DOI:** 10.1371/journal.pone.0037677

**Published:** 2012-05-25

**Authors:** Peter H. Wu, Steven Coultrap, Chelsea Pinnix, Kurtis D. Davies, Ramesh Tailor, Kian K. Ang, Michael D. Browning, David R. Grosshans

**Affiliations:** 1 Department of Psychiatry, University of Colorado Denver, Anschutz Medical Campus, Aurora, Colorado, United States of America; 2 Department of Pharmacology, University of Colorado Denver, Anschutz Medical Campus, Aurora, Colorado, United States of America; 3 Division of Radiation Oncology, The University of Texas M. D. Anderson Cancer Center, Houston, Texas, United States of America; National Cancer Institute, United States of America

## Abstract

Every year, nearly 200,000 patients undergo radiation for brain tumors. For both patients and caregivers the most distressing adverse effect is impaired cognition. Efforts to protect against this debilitating effect have suffered from inadequate understanding of the cellular mechanisms of radiation damage. In the past it was accepted that radiation-induced normal tissue injury resulted from a progressive reduction in the survival of clonogenic cells. Moreover, because radiation-induced brain dysfunction is believed to evolve over months to years, most studies have focused on late changes in brain parenchyma. However, clinically, acute changes in cognition are also observed. Because neurons are fully differentiated post-mitotic cells, little information exists on the acute effects of radiation on synaptic function. The purpose of our study was to assess the potential acute effects of radiation on neuronal function utilizing *ex vivo* hippocampal brain slices. The cellular localization and functional status of excitatory and inhibitory neurotransmitter receptors was identified by immunoblotting. Electrophysiological recordings were obtained both for populations of neuronal cells and individual neurons. In the dentate gyrus region of isolated *ex vivo* slices, radiation led to early decreases in tyrosine phosphorylation and removal of excitatory N-methyl-D-aspartate receptors (NMDARs) from the cell surface while simultaneously increasing the surface expression of inhibitory gamma-aminobutyric acid receptors (GABA_A_Rs). These alterations in cellular localization corresponded with altered synaptic responses and inhibition of long-term potentiation. The non-competitive NMDAR antagonist memantine blocked these radiation-induced alterations in cellular distribution. These findings demonstrate acute effects of radiation on neuronal cells within isolated brain slices and open new avenues for study.

## Introduction

Radiation therapy is used extensively to treat primary brain tumors and metastases and to prevent intracranial relapse in many malignancies. Successful treatment outcomes are, however, often tempered by a progressive decline in cognitive function, observed both in adults and children treated with cranial radiation [1], [2], [3], [4], [5]. Despite decades of research, little insight into the mechanism of cognitive dysfunction has been gained and no effective treatments exist.

Historically, radiation-induced injury to normal tissue has been attributed to DNA damage and the subsequent death of replicating cells [6]. As such cognitive impairment following brain radiation was viewed as irreversible. Alternative lines of evidence now suggest that surviving cells play an important role in long-term cognitive dysfunction following radiotherapy. Recent work in a rodent model has shown that transient hypoxia after whole brain radiation may reverse learning deficits [7]. Early phase clinical studies also suggest that when agents normally used to treat Alzheimer’s type dementia are administered following cranial radiation, they improve cognitive function [8]. These observations suggest that radiation-induced brain damage is perpetuated by surviving cells and potentially reversible.

Relatively little is known about the biologic mechanisms of cognitive decline following cranial radiation, but intriguing possibilities lie in our much-improved understanding of how neurons communicate, the molecular pathways involved, and the cellular processes believed to underlie cognition. The central nervous system is a dynamic environment in which neurons interact and alter their functions based on these interactions. Synaptic plasticity refers to the concept that brief periods of use provoke long-lasting changes in synaptic efficacy. One form of synaptic plasticity, in which brief high-frequency stimulation leads to long-lasting increases in synaptic efficacy, is known as long-term potentiation (LTP) [9], [10]. In the hippocampus NMDA (N-methyl-D-aspartate) and AMPA (α-amino-3-hydroxy-5-methyl-4-isoxazolepropionic acid) receptors mediate excitatory signaling. GABA (gamma-aminobutyric acid) receptors are responsible for inhibitory neurotransmission [10]. NMDA receptors play a unique role, directing changes in plasticity [11]. Neuronal plasticity has been the subject of intense study not only because it is widely believed to underlie learning and memory but also because the underlying processes may be involved in pathological situations [12], [13].

While patients treated with brain radiation frequently display long-term deficits in learning and memory, acute changes in cognition are observed clinically [14]. These changes are frequently overlooked and the molecular basis is unknown. Laboratory studies of oncologic models have shown that radiation leads to acute changes in tyrosine phosphorylation [15]. In neuronal cells phosphorylation-dependent trafficking of neurotransmitter receptors to and from the cell surface is a powerful regulator of synaptic strength and function [10]. Specifically, tyrosine phosphorylation-dependent trafficking of excitatory NMDARs to the synapse plays a role in LTP [16]. Conversely, tyrosine phosphatase-dependent NMDAR endocytosis may play a role in neuronal dysfunction induced by amyloid beta exposure [17]. We hypothesized that radiation, through early effects on phosphorylation, and thereby, localization of neurotransmitter receptors, acutely affects synaptic transmission. To test this hypothesis, we irradiated isolated acute and cultured rat hippocampal slice preparations. We show that radiation led to acute changes in NMDA and GABA transmission, findings that may underlie the acute changes in cognition that follow brain radiation.

## Results

### Radiation Alters NMDAR Phosphorylation and Differentially Affects NMDA and GABA_A_R Localization

**Figure 1:**
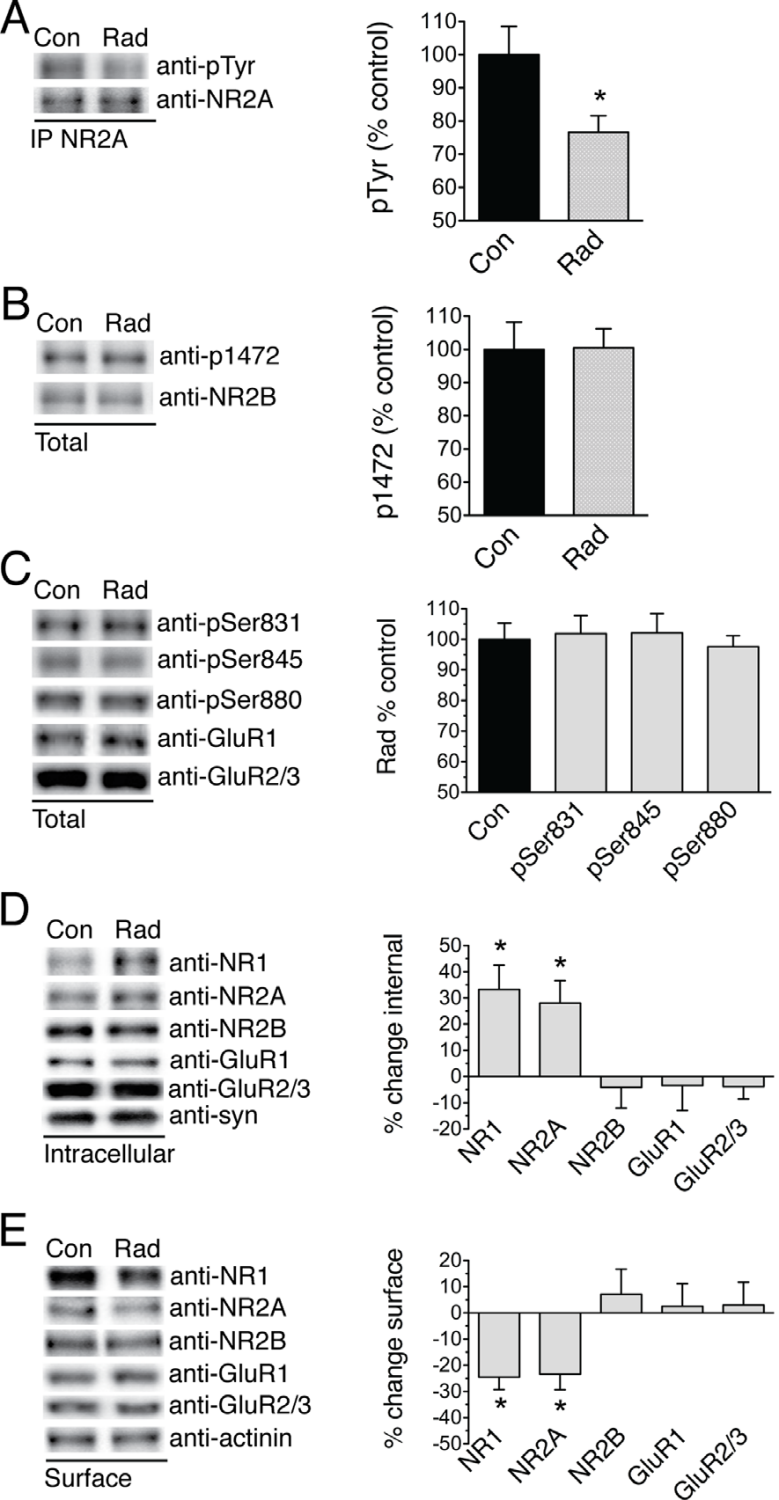
Radiation induces early alterations in NMDAR phosphorylation and internalization of NR1 and NR2A subunits. (A) Immunoprecipitation of NR2A subunits and semi-quantitative western blotting analysis of NR2A expression and phospho-tyrosine performed on dentate samples collected 30 minutes following a dose of 10 Gy radiation revealed diminished tyrosine phosphorylation. (76.7±4.9% of control, *p = 0.005, n = 7, data presented as mean ± s.e.m.) (B) No alteration in tyrosine phosphorylation at residue 1472 of the NR2B subunit was detected by western blotting from homogenates of dentate slices. (100.5±5.8% of control, p = 0.51, n = 6) (C) AMPA receptor phosphorylation status was assayed by semi-quantitative western blot analysis using phosphorylation site-specific antibodies. (GluR1; phospho-serine831 101.9±5.9% of control, phospho-serine845 102.1±6.3%, GluR2; phospho-serine880 97.6±3.6%, p > 0.4 for each, n = 8) (D) Sham and radiated dentate slices were incubated at 4°C in cross-linking reagent and levels of internal receptors quantified by western blot analysis of lower molecular weight bands corresponding to intact subunits. (NR1, 33.3±9.3% increase in intracellular, NR2A, 28.1±8.6%, *p = 0.004 and 0.005 respectively, students paired t-test with post-hoc Bonferroni correction; NR2B −4.1±7.9%, GluR1 −3.4±9.5%, GluR2/3 −3.8±4.8%, p > 0.3 for each, n = 11) (E) Irradiated and sham cultured slices were collected and incubated at 4°C in membrane-impermeable biotin. Surface receptors were separated by pull down with neutravidin beads and resolved by western blot. (NR1, 24.5±4.8% decrease in surface, NR2A, 23.4±5.9%, *p = 0.01 and 0.03 respectively, n = 10; NR2B 7.1±9.6%, GluR1 4.9±8.7%, p>0.25, n = 10; GluR2/3 2.97±8.8%, p = 0.89, n = 5).

The dentate gyrus is known to harbor neuronal precursor cells and play a key role in memory formation. Thus, this region is of interest with regard to radiation effects. To assay for changes in neuronal function within this region, the dentate gyrus was isolated from acutely prepared hippocampal slices and subjected to 10 Gy. Thirty minutes following radiation, dentate slices were homogenized and NR2A subunits isolated by immunoprecipitation. Tyrosine phosphorylation, assayed by semi-quantitative western blotting with anti-phosphotyrosine antibody, was diminished following radiation (Fig. 1A). Probing cellular lysates with anti-pTyr1472 did not detect any effect on the NR2B subunit (Fig. 1B). We next assayed for alterations in AMPA receptor phosphorylation utilizing antibodies directed to sites known to affect GluR1 or GluR2 function [18], [19], [20]. Radiation did not alter AMPA receptor phosphorylation (Fig. 1C).

Tyrosine phosphorylation of NR2A is believed to play a role in synaptic plasticity and long-term potentiation by affecting receptor surface localization [16]. In order to assess the effects of radiation on the subcellular distribution of glutamate receptors, isolated dentate slices were harvested 30 min after radiation and treated with the membrane-impermeable cross-linking reagent Bis(sulfosuccinimidyl)suberate (BS3). Cross-linking of surface receptor subunits produces higher molecular weight aggregates allowing them to be separated from internal subunits by gel electrophoresis. Analysis of internal receptor levels following radiation revealed rapid internalization of the NR1 and NR2A subunits of the NMDAR (Fig. 1D). Neither the NR2B subunit of the NMDAR nor the GluR1 or GluR2 subunits of the AMPA receptor were affected by radiation. The NR1 subunit of the NMDAR is subject to alternative splicing, and splice variants are in turn subject to differential trafficking. Using antibodies specific to the alternative splice variants, we showed that C2prime-containing receptors were specifically internalized (C2prime: 28.1±2.9% increase in intracellular, p = 0.002, C2: −2.6±16.2% n = 7).

To provide a second measure of surface receptor levels, we utilized a membrane impermeable biotin reagent to directly separate surface receptors using neutravidin beads. Biotinylation techniques are commonly utilized in cultured systems. However, we found that in acutely prepared dentate slices, this technique did not efficiently isolate surface receptors. As such, we utilized cultured whole hippocampal slices for biotinylation studies. The assay showed that levels of surface NR1 and NR2A subunits were decreased (Fig. 1E). While cultured slices were inclusive of dentate, CA1 and CA3 regions, this finding supported results obtained with cross-linking in acutely prepared dentate slices. To be certain that the biotin and BS^3^ modified only extra-membranous receptors, we tested the effects of these treatments on the intracellular proteins synapsin and α-actinin. These were unaffected by either technique. No effect of radiation on total protein expression levels of any target was seen at 30 min following radiation.

**Figure 2:**
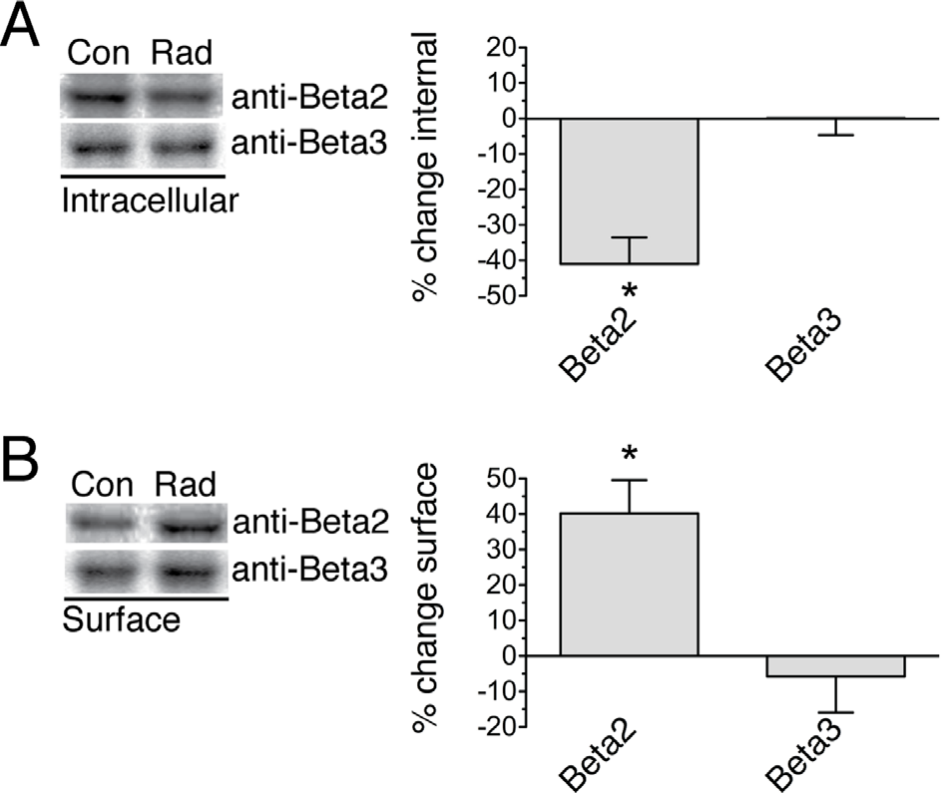
Radiation increases surface expression of GABA_A_ Beta 2 containing receptors. (A) Membrane-impermeable cross-linking studies, performed in dentate slices, revealed a decrease in the intracellular pool of Beta2 subunits without a detectable change in the localization of Beta3 containing receptors. (Beta2, 41±7.5% decrease in the intracellular pool of receptor, *p = 0.003, n = 9, Beta3, 0.2±4.8%, p = 0.6, n = 12) (B) Biotinylation of whole cultured hippocampal slices confirmed increased surface retention of GABA receptors containing Beta 2 subunits. (Beta2, 40.2±9.4%, *p = 0.0008, n = 9, Beta3, −5.78±10.16% n = 6, p = 0.9).

Similar to NMDARs, the activity of GABA_A_Rs is regulated by trafficking to and from the cell surface [21]. Differential trafficking of excitatory and inhibitory receptors has been reported [22]. Using both biotinylation and cross-linking techniques, we found that radiation increased surface expression of beta 2 containing GABA_A_Rs at 30 min, without effect on the beta 3 subunit (Fig. 2A and B).

**Figure 3:**
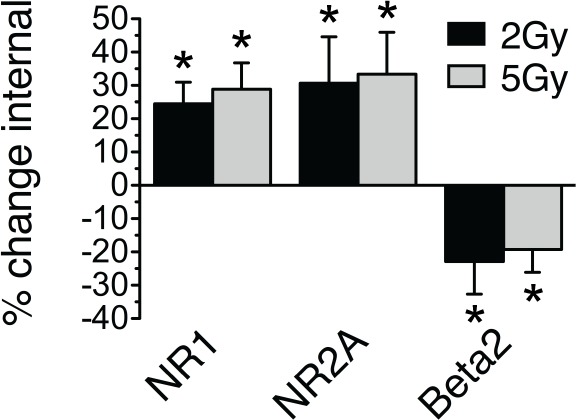
Receptor trafficking is observed following therapeutic doses. Dentate slices were incubated at 4°C in cross-linking reagent 30 minutes following sham, 2 Gy or 5 Gy radiation and internal receptors quantified by western blot. (NR1, 24.4±6.6% and 28.9±7.9% increase in intracellular 2 and 5 Gy respectively, NR2A, 30.7±13.9% and 33.4±12.6%, Beta2, −22.9±9.8% and −19.3±6.8%, *p≤0.05, n = 4 animals).

Because the 10 Gy dose is higher than the typical fractionated doses used clinically, we exposed dentate slices to more typical single-fraction doses of 2 or 5 Gy. Trafficking of NMDA and GABA_A_R subunits was consistently observed at both dose levels (Fig. 3).

**Figure 4:**
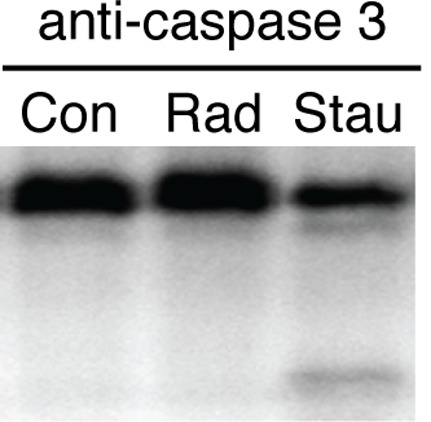
Radiation is not associated with caspase 3 activation. Whole hippocampal slice cultures were subject to sham, 10 Gy or staurosporine (Stau) and harvested at 1.5, 6 and 12 hours. Western blotting with anti-caspase 3 did not reveal evidence of caspase cleavage following radiation (12-hour results shown). Staurosporine treatment induced caspase cleavage and appearance of a lower molecular weight band.

Radiation may lead to apoptotic death of very young neurons [23]. This radiation induced neuronal apoptosis is associated with caspase 3 activation [23]. In contrast to acute slices, cultured slices prepared from young animals, may be maintained in culture for an extended period allowing for late measures, when apoptosis would be expected to peak. As such we measured caspase 3 activation in cultured slices harvested 1.5, 6 and 12 h after 10 Gy radiation. Staurosporine, a potent inducer of apoptotic death, led to caspase 3 cleavage and activation (Fig. 4). In slices exposed to 10 Gy, no caspase cleavage was detected at any time point, indicating that neurons survived this initial insult.

### NMDAR Internalization is Tyrosine Phosphatase Dependent and Prevented by Memantine

**Figure 5:**
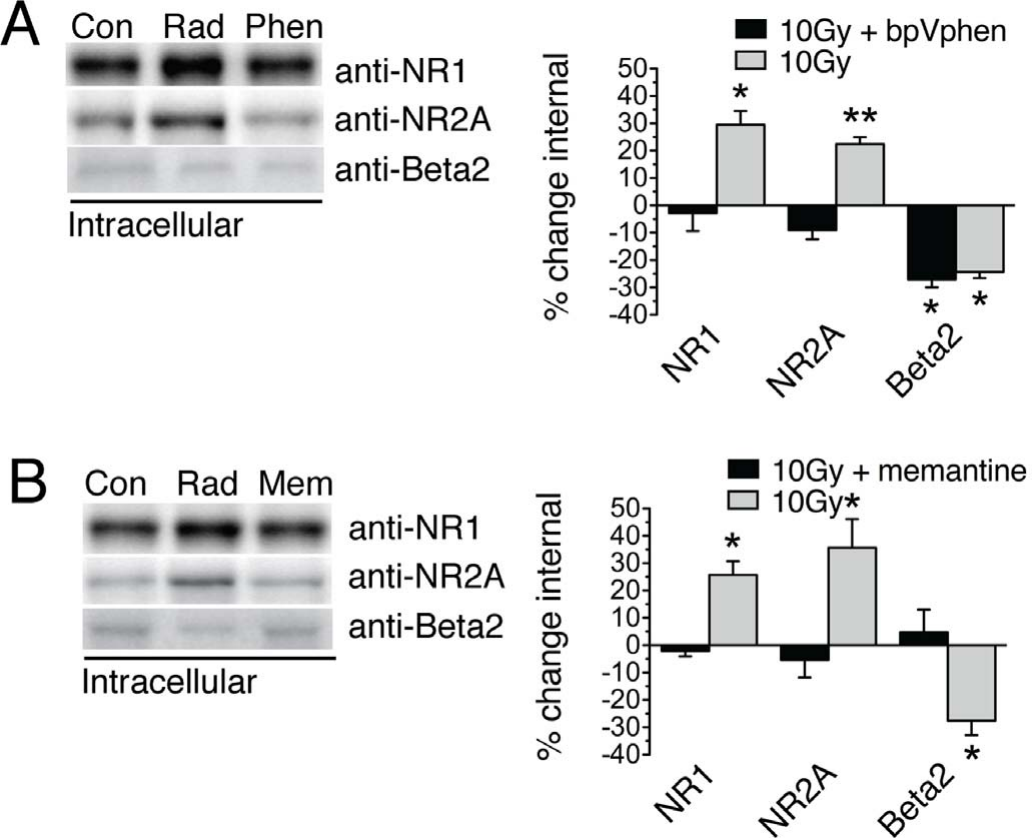
Radiation-induced NMDAR endocytosis is tyrosine phosphatase dependent and differential trafficking of NMDA and GABA receptors inhibited by memantine. (A) Prior to radiation slices were incubated in the tyrosine phosphatase inhibitor bpV(phen) (10 µM). Pretreatment was sufficient to prevent internalization of NMDA subunits, but had no effect on trafficking of Beta 2 subunits of the GABA_A_ receptor (NR1, −2.7±6.7% and 29.5±5.0% change internal 10 Gy with drug and 10 Gy respectively, NR2A, −9.1±3.3% and 22.4±2.6% Beta2, −27.1±2.3% and −24.3±2.2%, *p≤0.05, **p = 0.006, n = 4). (B) Pre-incubation in the non-competitive NMDAR antagonist memantine (50µM) prevented both NMDAR internalization and GABA surface retention (NR1, −2.1±2.0% and 25.7±5.0% change internal 10 Gy with drug and 10 Gy respectively, NR2A, −5.4±6.4% and 35.6±10.5%, Beta2, 4.7±8.4% and −27.5±5.3%, *p≤0.05 two-tailed Student’s *t*-tests, n = 4).

As mentioned earlier, tyrosine phosphorylation of NR2A subunits is believed to promote surface localization of NMDARs [16]. To extend this observation to the effect of radiation on tyrosine phosphatases as it relates to radiation-induced NMDAR internalization, we incubated dentate slices in the tyrosine phosphatase inhibitor bpV(phen) 20 min prior to 10 Gy radiation. Pretreatment was sufficient to prevent radiation-induced NMDAR internalization but did not affect changes in GABA_A_R localization (Fig. 5A).

To determine whether NMDA and GABA_A_R trafficking were dependent on NMDAR activity, we incubated dentate gyrus mini-slices 20 min prior to radiation with 50 µM memantine, an NMDAR antagonist approved for the treatment of Alzheimer’s dementia. Memantine blocked alterations in both NMDA and GABA_A_R surface expression (Fig. 5B).

### Radiation Alters NMDA and GABA Responses and Acutely Inhibits LTP

**Figure 6:**
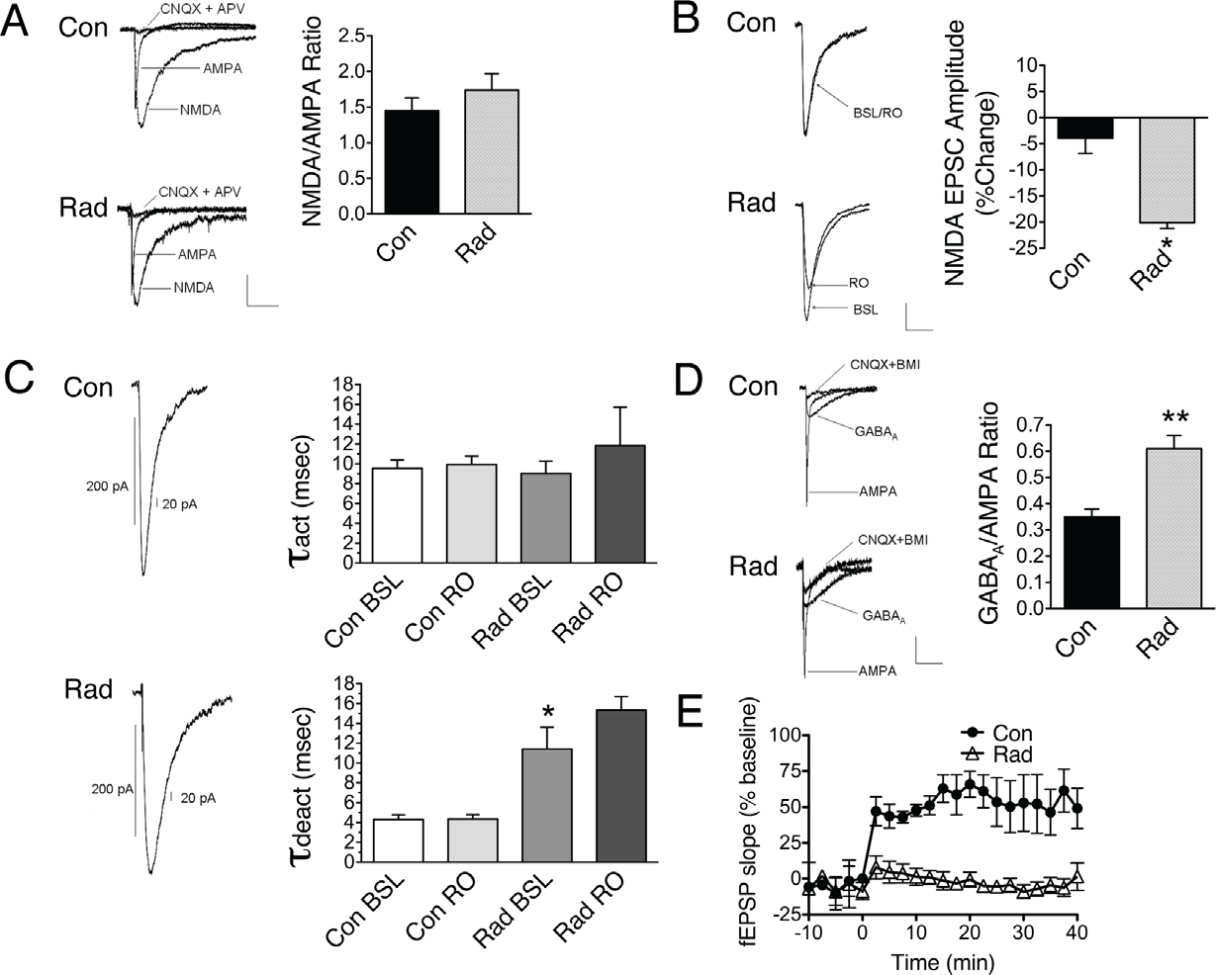
Radiation induces early alterations in synaptic function. (A) Whole cell recordings were obtained from dentate slices subjected to 10 Gy or sham. To allow for comparison between control (Con) and radiated slices (Rad), NMDA responses were normalized to AMPA responses. Initial recordings indicated radiation did not significantly change the amplitude of NMDA currents (p = 0.349, Student’s t-test; sham n = 7; rad n = 8). (B) However, NR1/NR2A responses obtained in the presence of the NR2B antagonist Ro25-6981, were significantly diminished in amplitude in radiated slices (*p<0.001, con n = 7; rad n = 8). (C) While average channel activation time (τ_act,_ the time, measured in msec to activate 200 pA current) did not differ between in control and radiated slices [F(3,32) = 0.361, p>0.782, one-way ANOVA], the average channel deactivation time (τ_deact,_ msec to inactivate 20 pA current) was significantly increased in NMDAR currents measured from radiated slices [F(3,32) = 13.751, **p<0.001, one-way ANOVA]. Ro25-6981 (RO) did not significantly alter τ_act_ or τ_deact_ of NMDA EPSCs in either control or irradiated slices. (D) GABA_A_ responses were normalized to AMPA responses. 10 Gy significantly increased the GABA_A_/AMPA ratio (**p = 0.001, con n = 6; rad n = 4). (E) In order to evaluate for potential changes in synaptic plasticity, baseline field EPSP recordings were obtained in control or radiated slices and LTP was induced by tetanic stimulation (HFS at time  = 0). In comparison to control (n = 4), LTP was significantly attenuated in radiated slices (n = 5, p = 0.02, 30 minutes post HFS).

To assay the functional status of receptors, we performed whole-cell patch recordings on sham or radiated dentate slices. Surprisingly, isolated NMDA responses were not reduced following radiation (Fig. 6A). However, NR1/NR2A-mediated responses isolated in the presence of the selective NR2B antagonist Ro25-6981 were significantly reduced (Fig. 6B). To further investigate the functional status of NMDARs, we analyzed channel kinetics. Recombinant NMDA receptors containing NR2A subunits have been shown to have faster deactivation kinetics (τ_deact_) than those containing NR2B subunits [24]. Irradiation of dentate slices did not affect activation kinetics (τ_act_) of NMDARs. However, radiation did significantly increase τ_deact_, consistent with a loss of NR2A-containing receptors or a proportional increase in NR2B signaling (Fig. 6C). We next measured GABA_A_R function. Further supporting the functional significance of the biochemical data, GABA_A_R mediated responses were elevated following radiation (Fig. 6D). Given the importance of activation of NR2A-containing receptors in the induction of LTP [25], we next examined LTP in dentate slices exposed to 10 Gy. Extracellular recordings from these slices revealed early attenuation of LTP (Fig. 6E), consistent with a loss of synaptic NMDA receptors and increased inhibitory signaling.

## Discussion

Radiation damage to normal tissues was historically attributed to DNA damage and the loss of proliferative cells. Because neurons are differentiated, non-proliferative cells, they were classified as radiation-resistant, and little attention was paid to the effects of radiation on synaptic function. The data presented here demonstrate that within the dentate gyrus region of isolated brain slices, radiation has acute effects on neuronal function.

Multiple lines of investigation now indicate that surviving cells play a role in cognitive dysfunction following brain radiation [26], [27]. Moreover, several lines of work more directly implicate abnormal neuron activity in long-term radiation induced brain dysfunction. Investigators studying the role of neuronal stem cells in learning and memory have utilized radiation as a tool to deplete proliferative cells and in doing so have documented long-term radiation effects on synaptic function, including diminished LTP [28]. Shi et al. undertook the first direct study of the long-term effects of radiation on glutamate receptor expression [29]. In adult animals, spatial learning was impaired 12 months after fractionated whole-brain radiation, and an increase in NR1 and NR2A subunits of the NMDAR was identified. Shi’s study implicates abnormal glutamate receptor composition in long-term memory dysfunction. However, the cellular localization and functional status of these receptors remains to be determined.

In contrast to studies of the long-term effects of radiation on brain function, few have focused on the acute effects on synaptic transmission at clinically relevant doses and none have assayed for early alterations in plasticity [30]. Utilizing biochemical assays we found evidence for a rapid radiation-induced differential trafficking of excitatory NMDARs and inhibitory GABA_A_Rs to and from the cell surface. The observed biochemical changes corresponded with impaired LTP induction. In whole cell recordings we noted that while pharmacologically isolated NR2A mediated currents were reduced, isolated NMDA responses were not. In addition to trafficking to and from the cell surface it believed that NMDARs are able to diffuse laterally into and from the synaptic cleft [31]. Neither crosslinking nor biotinylation are able to differentiate synaptic and extra-synaptic receptor pools. One hypothesis is that following irradiation, and internalization of NR2A containing receptors, there is a lateral diffusion of NR2B containing receptors into the synapse.

The mechanisms by which radiation induces differential receptor trafficking remain to be determined. Equally as important, relationship between early changes in synaptic function and long-term cognitive decline is unclear. However, potential mechanisms by which acute neuronal hypo-function might contribute to long-lasting brain dysfunction exist. Pharmacologic inhibition of synaptic activity produces dramatic, long-term changes in the composition of the post-synaptic density [32]. Specifically, diminished synaptic NMDARs may favor continued synaptic depression [33]. Thus, early radiation-induced neuronal hypofunction may lead to a persistent functional reorganization of the synapse. Neuronal activity also influences the survival and differentiation of neuronal precursor cells [34], [35]. Excitatory neuronal activity prompts precursor cells to adopt a neuronal fate [34]. It has been shown that a portion of stem cells survive radiation but are prompted to differentiate into glia by radiation-induced changes in the microenvironment [36]. Thus, if neuronal hypofunction persists following radiation this would, in theory, predispose to neuronal progenitor cells to adopt a glial rather than neuronal fate.

We found that application of the NMDAR antagonist memantine during radiation was sufficient to prevent changes in receptor localization. This both implicates abnormal glutamatergic signaling and provides a potential therapeutic strategy for protecting neurons from acute radiation damage. The long-term clinical benefits of memantine are currently being studied in a large randomized, multi-institutional trial, which recently closed to accrual.

The current study, performed in isolated brain slices, does not definitively link acute changes in neuronal activity to chronic cognitive dysfunction following radiation exposure. Moreover, the underlying mechanisms of differential receptor trafficking in response to radiation remain to be elucidated. However, the early changes in synaptic function we identified may underlie acute changes in cognition observed clinically. Moreover, given the important role of synaptic activity in directing cellular fate and function, the possibility that early alterations in synaptic function play a role in long-term radiation-induced cognitive decline exists. Future studies will address the mechanisms of differential receptor trafficking as well as seek to link early changes in synaptic dysfunction with long-term behavioral deficits.

## Materials and Methods

### Preparation of Hippocampal Slices

All animal studies were performed in accordance with NIH guidelines and approved by the University of Texas MD Anderson Cancer Center and University of Colorado Institutional Animal Care and Use Committees.

Hippocampal sub-regions may have differential sensitivity to toxic insults. As such we chose to focus on radiation effects within the dentate gyrus. The dentate plays an important role in learning and memory and frequent studied in regards to radiation effects, given the presence of neuronal stem cells. For all experiments utilizing acute slices, the dentate gyrus was isolated from acutely prepared whole hippocampal slices. Whole slices were prepared from 12- to 15-day-old Sprague-Dawley rats and two cuts employed in order to isolate the dentate as previously described [37]. Following a 90-minute recovery period, these isolated dentate slices were utilized for experimentation.

Hippocampal slice cultures, inclusive of dentate, CA1 and CA3 areas were utilized only in biotynylation studies (detailed below) and for late measures of caspase activation. In order to ensure viability, hippocampal slice cultures were prepared from postnatal day 6 to 7 Sprague-Dawley rats using sterile conditions and maintained in culture for 7 days prior to radiation. The brain was removed and placed in dissecting solution (87 mM NaCl, 2.5 mM KCl, 7 mM MgCl_2_, 0.5 mM CaCl_2_, 1.25 mM NaH_2_PO_4_, 25 mM glucose, 75 mM sucrose and 25 mM NaHCO_3_); and 400-µm thick hippocampal slices prepared. Slices were maintained using an interface technique [38]. The initial culture medium consisted of 50% Opti-MEM with glutamax, 25% horse serum, and 25% Hanks’ solution with penicillin and streptomycin. Following 3 days in culture slices were transitioned to medium containing Neurobasal A with 2% B27 supplement, glutamax, dextrose, and penicillin/streptomycin. Half of the culture medium was replaced every 3 days.

For drug experiments, the drug was applied 20 min prior to radiation, and slices were maintained in memantine or bpV(phen) until harvest.

### Irradiation

Irradiation was performed using a RadSource RS2000 orthovoltage tissue irradiator, 160 kVp, half-value layer 1.1 mm Cu or Co-60 source. For each unit, slices were positioned to receive 1 Gy/min. Radiation doses were confirmed in initial experiments for each source using thermoluminescent dosimeters. All controls were prepared in the same way and subjected to sham radiation.

### Analysis of Neurotransmitter Receptor Surface Expression

We measured surface expression of excitatory and inhibitory neurotransmitter receptors using membrane impermeable cross-linking, as has been described in detail [39]. For biotinylation experiments cultured slices were utilized in place of acute dentate mini slices, as biotinylation was not reliable in acute preparations. Following radiation, whole cultured slices were harvested and rinsed with cold artificial cerebrospinal fluid (ACSF) and incubated for 1 h in ACSF containing 1.5 mg/ml sulfo-NHS-SS-biotin (Pierce) on ice. Slices were subsequently rinsed in ACSF containing 20 mM Tris, pH 7.6, to quench free biotin and sonicated in 0.1% SDS, 1% Triton X-100. 25% of the homogenate was removed and sample buffer added to it. The remaining homogenate was incubated at 4°C overnight with NeutrAvidin beads (Pierce). Beads were rinsed three times with ACSF and resuspended in sample buffer.

### Semi-quantitative Western Blotting and Immunoprecipitation

Immunoprecipitation and semi-quantitative western blotting were performed as detailed previously [16], [37]. Antibodies to synapsin, GluR2/3, NMDA and GABA_A_R subunits and ones to specific AMPA and NMDA receptor phosphorylation sites were obtained from PhosphoSolutions. Anti-GluR1 and α-actinin were purchased from Chemicon. Blots were developed in SuperSignal chemiluminescent substrate and images acquired and quantified using an Alpha Innotech system.

### Electrophysiology Experiments: Fields and Whole Cell Patch

Field excitatory postsynaptic potentials (fEPSPs) were elicited with a bipolar tungsten-stimulating electrode and recorded using a silver electrode within a drawn glass capillary filled with ACSF. Dentate gyrus fEPSPs were generated by stimulation of the medial perforant path and recordings obtained in the molecular layer. Following 20 min of stable recording, input/output curves were generated. Stimulation intensities producing half-maximal response were chosen for test pulse, and baseline recordings were obtained. To induce LTP, we delivered four sequential 1-s trains of 100 Hz, separated by 30 sec each.

Whole-cell recordings of granule cell neurons in the dentate gyrus were obtained using patch pipettes with 6 to 10 MΩ resistance, filled with internal solution, which consisted of 140 mM CsCl, 2 mM MgCl_2_, 1 mM CaCl_2_, 10 mM EGTA, 10 mM HEPES, 2 mM NaATP, and 5 mM QX-314, 295 mOsm, pH 7.29 using CsOH. Pharmacologically isolated AMPA receptor-mediated excitatory postsynaptic currents (AMPA EPSCs) were obtained by recording signals from the cell in the presence of the GABA_A_ receptor blocker bicuculline methiodide (BMI, 20 µM), GABA_B_ antagonist 3-[[(3,4-dichlorophenyl)methyl]-amino]propyl]-diethoxymethyl)phosphinic acid (CGP 52432, 1 µM) and the NMDAR blocker D-(-)-2-amino-5-phosphonopentanoic acid (APV, 50 µM).This isolated current was completely blocked by 6-cyano-7-nitroquinoxaline-2,3-dione (CNQX, 20 µM). NMDA EPSCs were recorded in the presence of CNQX, BMI, and CGP in 0.5 mM Mg^2+^ ACSF. GABA_A_ currents were obtained in the presence of CNQX, CGP and APV. To evoke a synaptic response, the stimulating electrode was placed 300 µM from the recoded cell in the inner blade dentate gyrus. The input-output relationship was established for AMPA EPSCs up to the maximum stimulus strength that produced an action potential. Recordings for AMPA EPSCs, NMDA EPSCs, and GABA_A_ IPSCs of each neuron were measured at 70–80% of the maximum stimulus. In some experiments, Ro25-6981 (1 µM), a selective NR2B antagonist, was perfused onto the slice to inhibit NR2B subunit-containing responses. τ_act_ and τ_deact_ were calculated according to the modified methods reported by von Engelhardt et al [40].

### Statistical Analyses

Except where noted, data were analyzed with paired, two-tailed Student’s *t*-tests with post-hoc Bonferroni corrections to account for multiple comparisons. All *n* values refer to the number of animals used and each experiment was independently performed a minimum of four times.
